# Strengthening Health Systems Using Innovative Digital Health Technologies in Africa

**DOI:** 10.3389/fdgth.2022.854339

**Published:** 2022-03-31

**Authors:** Sunny Ibeneme, Humphrey Karamagi, Derrick Muneene, Kajali Goswami, Noel Chisaka, Joseph Okeibunor

**Affiliations:** ^1^Harvard T.H. Chan School of Public Health, Boston, MA, United States; ^2^World Health Organization – Africa Regional Office, Brazzaville, Congo; ^3^World Health Organization, Geneva, Switzerland; ^4^World Bank Group, New York, NY, United States; ^5^World Bank Group, Maseru, Lesotho

**Keywords:** Africa, Digital Health Platform, health systems strengthening, Universal Health Coverage, Sustainable Development Goals

## Abstract

While effective health systems are needed to advance Universal Health Coverage and actualize the health Sustainable Development Goals, information system verticalization remains a challenge among African health systems. Most investments are vertical, partner-driven and program-specific with limited system-wide impacts. Poor linkages exist amongst different solutions as they are not designed to capture robust data across multiple programmatic areas. To address these challenges, the World Health Organization Africa Regional Office has proposed the adoption of a Digital Health Platform (DHP) to streamline different solutions to a cohesive whole. The DHP presents a pragmatic approach of bringing multiple platforms together using recognized standards to create a national infostructure, which bridges information solutions toward healthy and sustainable outcomes. It has capacities to curate accurate, high fidelity and timely data feedback loops needed to strengthen and continuously improve program delivery, monitoring, management, and informed decision-making at every level of the health system regardless of location. This paper contributes to the ongoing regional conversations on the need to harness innovative digital solutions to improve healthcare delivery in Africa.

## Introduction

Discussions on harnessing innovative ways of bridging the digital divide among African health systems have continued to gain traction over the past decade. Current challenges with digital solutions in Africa limit their overall impact as most solutions are institution-specific with limited system-wide impacts (1). There is limited emphasis on system strengthening to drive sustainable developments in countries. Most health investments are vertical, partner-driven and program-specific. Partners too often adopt the reductionist perspective that places more focus on disease priorities, with the belief that the system will be strengthened when interventions are prioritized for specific diseases (2). There is limited policy buy-in by stakeholders, as partners often seek to produce quick results for the Sustainable Development Goals (SDGs) through fragmented siloed programs. There are also poor linkages amongst different solutions as they are not designed to capture robust data across multiple sectors and programmatic areas. While most solutions lack mechanisms to capture high fidelity real-time data to respond to current needs; others are not designed to support interoperability and data sharing across the continuum of care (1).

These are fragmented, inefficient vertical silos systems built with minimal involvement of end-users. In addition, there is low management and governance capacity to coordinate digital health solutions that are responsive to regional needs (3). These peculiar challenges result in major gaps in the ability of digital health solutions to respond to the needs of Member States (4, 5). According to the WHO Regional Committee report, different Member States are at different digital health maturity level, which could impact the provisions of integrated care and systemic healthcare digitization (6, 7). The Global Observatory report of 2015 on eHealth documented varied level of use of different digital solutions among African health systems, with mHealth technologies reported as the most used innovation, and big data documented as the least used innovation (6).

This article aims to contribute to the ongoing regional conversations on the need to harness innovative digital solutions to improve healthcare delivery in Africa. The paper highlights key challenges, ongoing progress and future directions in-view of the use of the World Health Organization African Regional Office (WHOAFRO) Digital Health Platform (DHP) to strengthen African health systems for the SDGs. It explores approaches for operationalizing the WHOAFRO DHP, and proposes policy recommendations for addressing persistent digital divide among most African economies. The adoption and institutionalization of the DHP among African health systems has opportunities to bridge information solutions toward healthy and sustainable outcomes in countries.

As a key enabler of health information across the spectrum of data, analytics, and knowledge; digital health solutions contribute to the strengthening of health systems as countries move toward the achievement of the Universal Health Coverage (UHC) and the health Sustainable Development Goals (SDGs). Given the appropriate infrastructure, skills, and education, digital technologies have opportunities to improve access to essential healthcare services, while reducing paper-based reporting systems. When fully optimized and functional, integrated digital solutions have opportunities to positively impact sustainable health outcomes including agreed sustainable development goals and targets (3, 4).

The WHOAFRO DHP is a facility-wide end-to-end digital solution that enables users to collect, analyze and interpret clinical information. The platform can be manipulated to fit the hospital workflows and can be customized to fit Member States' health systems across different service delivery levels. The platform has several modules that enable users to easily enter, retrieve and analyze data within the facility, based on their needs, roles and responsibilities. Thereby, enabling the users to: Track data over time; Identify patients who are due for preventive visits and screenings; Monitor how patients measure up to certain parameters (e.g., vaccinations); and improve overall quality of care among others (1, 7).

## Digital System Fragmentation Creates System Inefficiencies

Advancements in digital technology witnessed among global communities brought opportunities and threats to health systems. By the end of 2018, more than two-third of the global community subscribed to a mobile service. The falling price of connectivity and the rollout of 5G networks have encouraged many more to adopt the innovative digital technology (5). The increasing digital solutions have opportunities to transform health systems from reactive to proactive to predictive systems that consolidate integrated care and sustainable developments in countries (7). This has led to increased access to reliable health information and has strengthened TB and HIV outcomes (8) as well as psychiatric outcomes (9) among others.

While innovative digital technologies have opportunities to bridge systems for sustainable developments in countries, studies show that digital fragmentation arising from systemic proliferation of systems have opportunities to exacerbate inefficiencies (10, 11). The rising pace of digitization poses threats to the digital health ecosystem as numerous digital solutions compete with each other with no systemic integrations for impacts. Most solutions are standalone and are deployed in response either to specific needs or to support specific programs, with significant gaps in collecting and analyzing real-time digital data (12, 13). The Ebola response in the Democratic Republic of the Congo presents a topical use-case where lack of connectivity, systemic integrations and coordination led to redundant efforts that mitigation efforts. Multiple vertical solutions deployed by partners without systemic integrations with national infostructure could not share real-time data, and lacked consistency in data entry and coding protocols. These decreased data quality and created opportunities for errors, thereby impacting surveillance mechanisms (14).

Siloed solutions led to increased burden on system users as well as overall medical practice errors (15). Gleiss and Lewandowski (15) reported an increased misdiagnosis, inappropriate medication dispensing and duplicate services following numerous standalone solutions. These introduced systemic inefficiencies as they require healthcare workers and system administrators to use multiple, unconnected digital applications iteratively. Healthcare worker were made to login into many applications with different access methods and identifiers to be able to do a particular work that could be essentially interrelated. The duplicated efforts introduced systemic confusion, data entry errors and staff burnout, and impacted the overall quality of service delivery (15).

These translate to constraints to innovation (16). Software developers spend time writing redundant codes for standalone applications that could be shared as common core technologies. According to WHO Reports (2019), this status quo impacts the development of an integrated national infostructure that connects multiple systems, and undermines governments' efforts on delivering quality health services (17). In addition, data security, privacy and identification issues remain a concern for both users and the government (10). The question pertinent is whether it is feasible to develop an integrated solution that could solve regional information solutions challenges. Yes, this is possible; the technology exists. The WHOAFRO DHP has opportunities to address identified challenges through robust planning, implementation, and review mechanisms including the establishment of regular reviews, communities of practice, and capacity improvements supported by technical experts at WHOAFRO including other implementing partners. Such mechanisms encourage governments to be accountable and committed as they are guided to prioritize deploying integrated solutions that serve the needs of the people and are linked to prioritized service outcomes (18).

## Integrated Response to Fragmented Digital Solutions Among African Health Systems

To address health systems' challenges and strengthen regional health systems for the SDGs, the WHOAFRO proposed the use of a comprehensive DHP to address peculiar regional digital challenges and provide integrated digital solutions that align with the needs of countries (1, 18). The WHOAFRO DHP is a facility-wide electronic health records system that ensures the realization of UHC pillars of “Accessibility, Quality and Affordability” and has opportunities to accelerate innovation at country levels. It enables individual applications and systems to interoperate and work together in an integrated manner for robust outcomes. The platform fosters interoperability through standard-based Health Information Exchange and health information architecture known as infostructure. DHP infostructure comprises a set of integrated common and reusable components, which are core technology services required by applications for the efficient running of digital health systems including registries, data repositories and identity authentications among others. It is one component of the complex systems that make up national health systems. Thus, with infostructure as part of the DHP, the influential impacts of governmental leadership on DHP institutionalization could be exponential, and have opportunities to impact its scope and scale (1, 2).

The DHP is an open-source, open-standard public good that provides robust digital solutions that facilitates comprehensive electronic management of patient health records. It supports ICT infrastructure principles for digital development and investment, as well as provides a framework for sustainable implementation and capacity-building (19). The platform corroborates other regional goods as it upholds the core principles and values of WHOAFRO's Action Framework, which highlights the interconnected nature of health systems and services through effective, equitable and efficient service delivery (18). The basic elements of the WHOAFRO DHP is represented thus:

The WHOAFRO DHP could provide a horizontal base digital solution that connects vertical siloed information systems including functional and non-functional requirements that are housed within individual digital health applications. Through DHP's interoperable standard-based design, exchange of information is facilitated efficiently whenever the need arises: All data passes through the DHP hub, whether they are stored on the DHP alone or divided among multiple external repositories and applications. Such information exchange occurs through DHP integrated services, authentication services, common workflows support services, consistent terminologies and reference data, and other components that help streamline and improve efficiency (1).

The WHOAFRO DHP enables users to collect, analyze and interpret clinical information (1). Being modular, the system can be manipulated to fit specific hospital needs and workflows. In addition, modules can be customized to specific needs of a given hospital business processes. The WHOAFRO DHP is an open source modular system for use by service providers to capture and record interactions with clients from entry to exit of a hospital (1). It is built as an extension of the open source OpenMRS (version 1.98 with the 2.0.4 UI Library Module). Frontend application architecture comprises ASP.NET, Bootstrap, XML, XSLT and Pooper, and it uses ICD-11 standards (20). The platform is built on a Model View Controller (MVC) design pattern; and on a framework which is an object-relational mapper (O/RM) that enables.NET developers to work with a database using.NET objects (20). It is designed to capture real-time service delivery and management events as they occur in a facility; Provide standard guidance to service providers during the process of care—for example standard clinical practice, use of ICD-11 for diagnosis, use of essential medicines list for drug management; Build a repository of health and management events occurring in facilities; Monitor adherence to clinical and management guidelines during provision of clinical and public services; Highlight real-time notifiable events as they are captured in the facilities; and Allow for information sharing across facilities (1, 2).

The scope of WHOAFRO DHP hardware requirements are dependent on the hospital. It is recommended to use existing hardware, but plan for medium to long term Information Technology (IT) improvements to maximize the use of the DHP. Equipment to consider include but not limited to: Computers, tablets, server point, single finger print reader (if biometric information is needed), printing services, power solutions, and communication and networking equipment, to allow the different service points be interlinked with each other, and to the server. It is preferable that both cabling, and an intranet wireless system are installed; though this would depend on country context and needs that should be determined following site assessments (1).

Potential products of the WHOAFRO DHP solution are pragmatic. In line with the needs of a hospital, the WHOAFRO DHP solution can be customized to provide the following: Modules of care based on the specific focus services of a hospital; Automated summary of records and analysis of data arising from the DHP data that is collected, in line with the specific needs of the hospital; An Application Program Interface (API) to link the DHP to any previous IT system the hospital was using, and so access past electronic records; A client portal, where patients can access and enter records for data they need to collect from home, and receive key information/messages and interact with their providers; A “How to” guide to facilitate self-training by providers in the installation and use of the DHP with limited need for external expertise; and Access to a wider DHP “Community of Practice” where issues and solutions can be discussed with a global pool of OpenMRS experts (1, 2).

Developed by the WHOAFRO, this platform is expected to evolve and mature over time depending on country needs, context and complexity. It begins with a core set of functionalities necessary to support initial digital health applications that any country wishes to integrate and evolve over time. More functionalities are added in-view of country DHP's maturity and sophistication to support more services and programs prioritized by countries. It has globalization flexibilities, as it can be translated into different languages per country needs and contexts. The DHP supports integrated service delivery through four perspectives including Health Providers' Portal, Health Managers' Portal, Community Statistics Portal and Personal User Portal.

**Figure 1:**
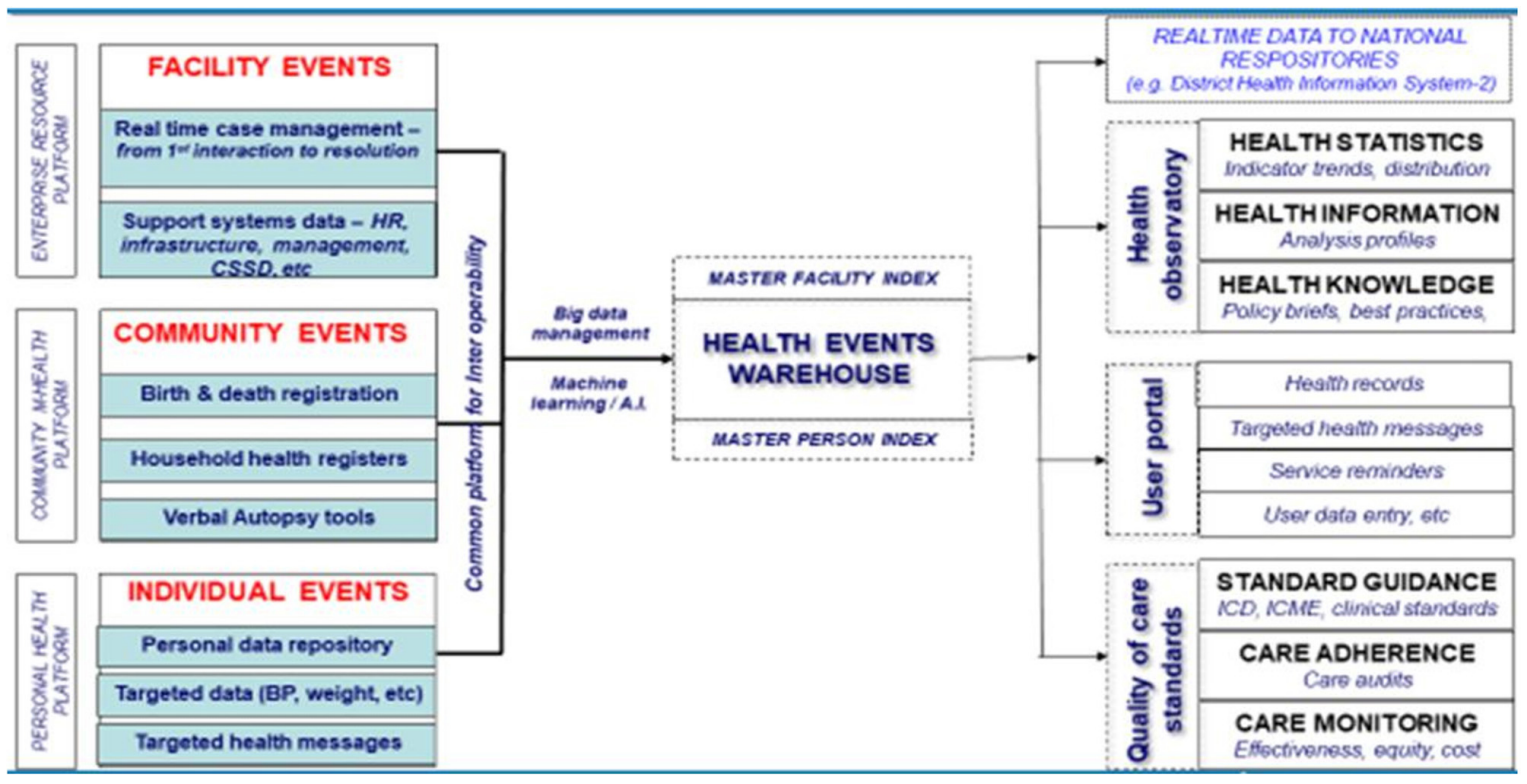
Comprehensive WHOAFRO Digital Health Platform elements.

Health providers' portal and health managers' portal are part of the enterprise resource platform and represent health facility events at the enterprise level (Figure 1). It documents real-time case management of health events from first interaction with the patient to resolution, as well as highlights relevant support systems that are available in health facilities including Human Resources for Health, Health Facility Management, and Central Sterile Service Department among others. Actions defined around this domain are mainly those related to health systems' building blocks including service delivery, health workforce, health infrastructure, medical products and health technologies. Others are health governance, health financing, and health information (1, 21). Performance under this domain is measured by health system resilience, efficiency and equity of access, quality of care, and service demand (18).

Community health platforms define points where health investments are made on the use of community statistics for routine monitoring, evaluation and surveillance. Such health investments are made in the form of data systems, hardware/software tools and community-support processes that enable community surveillance and translates to defined outcomes and impacts. Actions defined under this domain are mainly those related to birth and death registration, household registries, and verbal autopsy tools (1). Performance under this domain is measured by improvements in life expectancy, morbidity and mortality reductions, and risk factor reductions mainly at the individual and community levels (18).

Lastly, the personal user portal represents individual events at the patient and client-level, and comprises personal data repository, vital signs data (weight, height, temperature etc.,) and targeted health messages amongst others (1). Elements under this portal comprises: Availability of essential services for care users, coverage of essential services for target groups, client satisfaction, health financial risk protection, and cross-sector coverage of essential health services (18). Thus, clients and care-seekers are able to access information related to the care and services they need (22).

The DHP infostructure enables interoperability of the listed portals for system efficiency. Information and data systems from the portals pass through the data hub of the DHP to the data warehouse enabled by robust Master Facility Index and Master Person Index (Figure 1). Agile health big data systems, management and analytics enabled by Machine Learning (ML) and Artificial Intelligence (AI) applications facilitate programmatic data analytics for policymaking, system improvements and intervention mapping (23). This also facilitates the generation of real-time data on national repositories like the District Health Information System-2 platforms, and sends live information updates on national dashboards like national health observatories for health statistics (health indicators, trends and distribution), health information data (analyses profiles) and health knowledge (policy briefs and best practices). Others are live information updates on patient user portals for patient health records, targeted health messages, service reminders and user data entries among others. It also facilitates real-time updates on quality of care standards including standard guidance (ICD, ICME and Clinical standards), care adherence (care audits) and care monitoring (effectiveness, equity and cost) among others (1).

## Operationalizing the WHOAFRO Digital Health Platform: Consolidating Strategies for Integrated Regional Digitization

Overall, the DHP is more encompassing than the Electronic Health Record as it captures personal health records, and places the management of health within country systems as well as in the hands of health-seekers. It facilitates the attainment of UHC from healthier populations perspectives by ensuring that individuals are able to receive health services without any financial hardship. Through this holistic approach, need is addressed through the perspective of demand (and not merely supply) for services that would help populations achieve healthy lives and wellbeing. As low demand translates to low utilization, populations must be aware of what is available, accessible and useful for them. This will help in improving health-seeking behaviors, decisions and actions of individuals (12, 24). Proper community engagement, enlightenment and capacity-building on health are critical to the change management and knowledge translation processes involved in the successful implementation of the platform (1).

The adoption of the WHOAFRO DHP provides the opportunity to translate robust frameworks into operational strategies for countries to strengthen national health systems for the SDGs through effective digital approaches. The WHOAFRO DHP supports the development of an integrated health sector digitization strategy for Member States, which aligns and incorporates other national e-government initiatives for improved national outcomes. Digital Health Strategy creates a clear path that guides the investments of the Governments and their development partners. It outlines a time-bound, practical, sustainable, and cost-effective plan for the deployment of a set of integrated information systems that supports the achievement of national goals, targets and priorities (6).

The Estonian model highlights critical pathways the DHP connects with the e-government frameworks. The Ministry of Social Affairs, Estonia established a unified digital platform that linked public and private enterprises through efficient interoperable secured architectures. The platform leveraged on existing cross-institutional digital integrations of key governmental sectors including but not limited to e-taxation, e-banking and e-school through unique national electronic identifiers (1, 13). The Estonian model corroborated the mHero deployed in Liberia during the Ebola outbreak of 2014. The Ministry of Health (MoH) connected existing systems- human resources information systems (HRIS) with Short Message Service (SMS) through efficient RapidPro platforms that facilitated information exchange between health workers. This provided real-time information exchange harnessing OpenHIE frameworks for integrated disease surveillance and response (1). Thus, the mHero of Liberia, the FamilyConnect of Uganda and the Sistema Electronico de Logistica de Vacinas of Mozambique are other related digital platforms with integrated e-government frameworks and robust trust frameworks (19, 25).

Aerts and Bogdan-Martin documented the use of the Broadband Commission's framework for evaluating digital technology proliferations, including its strengths in identifying and addressing critical digital gaps (10). This corroborated the study by Moore et al. (13) which highlighted the use of an integrated digital health framework to design effective health information systems with system-wide impacts. They emphasized the need to consolidate technologies which are integrated, scalable and interoperable (11). Thus, the WHOAFRO envisages that the development of the regional DHP and its country-specific implementations will strengthen regional health systems for the SDGs. To achieve this, there will be a need to accelerate the shift from targeted solutions to interoperable system-wide solutions with enhanced system performance for improved health outcomes (1).

The implementation of the DHP is never linear in practice, but has areas of overlap of activities in accordance with the WHO Digital Implementation and Investment Guide (DIIG). Key implementation steps include: Conduct context analysis; Design and establish DHP architecture; and implement DHP by institutionalizing the DHP among national health systems. Countries implementing DHP are guided through a systematic approach of gathering functional and non-functional requirements for developing, designing and implementing meaningful digital health interventions in-line with recommendations from DIIG. However, system governance, local configuration needs and data hosting is at the country level/servers with technical support from WHOAFRO and other implementing partners (1, 2).

Thus, the DHP improves health systems at scale by fostering linkages and interconnections that dissuades redundancy and silos approaches to health system thinking (3). The DHP has mechanisms adaptable to differing settings that allow Member States to contextualize the solution based on their contexts, capacity and needs while allowing WHOAFRO and other implementing partners provide the necessary technical assistance. This includes providing guidance on technology selection, capacity development and Monitoring & Evaluation (M&E) frameworks as necessary. This helps appraise the DHP performance data, identifies training needs, facilitates identification of platform bottlenecks, and optimizes systems' performance. It also supports the identification of DHP's potential advancements, interventions and accomplishments, DHP infostructure design, its development stage and uptake by users (1).

The gap is a lack of integrated conceptualization of digital health needs. For instance, interoperability challenges are due to piecemeal digital solutions that do not talk to each other. Thus, emphasis should be on building and sustaining people-centered integrated solutions with system-wide impacts. The prevailing COVID-19 pandemic has reinforced the need to have integrated solutions given the range of possible digital needs. Countries are willing to adopt the DHP solution, but the challenge amidst many others, is in current technology vendors that develop and maintain independent systems (1). Thus, moving forward, evolving technologies of interest should be embraced including big data management, AI and ML innovations to improve AI-powered predictions, analytics and optimizations for sustainable health outcomes (26, 27).

## The Future of African Health Systems' Digitization: Bringing the Best of Technology to Health

As part of the global efforts to transform health, the WHOAFRO through effective mechanisms has continued to harness the power of innovative technology to support countries to achieve health SDGs including emergency preparedness and response. A key lesson learned is the importance of taking holistic approaches when developing digital health systems. Building standalone solutions should be discouraged including the existence of too many redundant solutions that conflict with one another. Countries should address this on time as they begin health system digitization discussions. They should move toward single, dominant modular home-grown systems that integrate all elements. Such systems should be open source and open standard systems (27). The DHP infostructure offers efficient foundations for cohesive systems, and is built and designed through holistic approaches that enables systems' expansion, integration and updates. Such solutions enable interoperability and are tailored to the needs of specific health systems involved (1).

Digital health applications and solutions should be government demanded, and not market-driven solutions. Governments are to map requirements according to needs and contexts, for which the digital health community can work concertedly to address prioritized national needs: It should not be the other way round. This exercise should be informed by countries' digital health strategy, standards, enterprise architecture and other regulatory protocols as necessary. Such governance mechanisms should focus on how to build and sustain integrated solutions in countries. Implementing partners are to provide support and guidance to the government on digital health strategy development, solution procurement and enterprise architecture (17, 26). This should conform to national infostructure which enables greater flexibility that facilitates reusability and interoperability with external systems through recognizable standards and APIs. Thus, this consolidates health systems efficiencies and improvements by allowing more national digital health interventions to plug in, thereby accommodating evolving digital technologies including AI, Internet of things and new medical technologies as necessary.

The eGovernment overarching architecture should always be adhered to, as this guides the overall government's expectations with e-solutions. It facilitates the linkage of solutions, and takes advantage of eGovernment support to ensure system integrations with existing national systems and repositories. Thus, vendors including implementing partners are encouraged to map existing solutions against country digital health standards, infostructure and enterprise architecture. Collaboration is key in digitization (21). Public private partnerships should always be consolidated for sustainability. It enables exchange of experiences for faster uptake of lessons learned. Cross-country collaborations for learning and sharing of experiences foster communities of practice roles and designs. This fosters efficient health systems' digitization of scale and scope. Such knowledge exchanges by digital health professionals have opportunities to leapfrog national health systems to new frontier technologies, while building competencies and skills to scale and sustain investments (21, 27).

In addition, the DHP could foster a culture of data use within the health sector. It could also be used to recognize burgeoning disease epidemics or community health trends by routinely tracking and analyzing service delivery data. Once identified, the various digital tools available on the DHP can be used to help address such events. DHP enables detailed understanding of different COVID-19 needs by population demographics, and provides real time progression of COVID-19 within a population. The platform supports real-time monitoring of medical interventions– ensures and monitors treatment standards, surveillance protocols, as well as collates data on treatment regimens and outcomes. There are interlinkages of different data to understand trends, distribution impacts, including prediction and attribution information at the individual and population levels. The platform also populates long-term follow-up data which are part of policy data to inform policy-making, intervention mapping and overall system improvements (28).

Lastly, with a digital health platform in place, African countries can embark on more successful digital health programs that can rely on built-in system infostructure to access important and reliable information; connect national/rural health facilities and workers to specialist care and higher-level professional training through well-coordinated telemedicine networks; improve health financing processes including fraud detection; as well as identify the users of the applications and data on the DHP infostructure. Patient care options could also be expanded by DHP enabling home-based monitoring and electronic health records and prescriptions that can be accessed and updated iteratively wherever patients seek services, no matter the device and location (1). Thus, the need to adopt and implement an integrated solution with robust regulatory frameworks cannot be over-emphasized.

## Conclusions

This paper contributes to the ongoing Regional conversations on the need to harness innovative digital solutions to improve healthcare delivery in Africa. The paper summarized topical information systems' challenges among African health systems, and enumerated the possible ways of addressing identified challenges using the WHOAFRO DHP, including how the platform could be operationalized to strengthen Regional health systems for the SDGs.

Notable challenges anticipated with implementing the WHOAFRO DHP include but limited to: High cost of software; Lack of in-country capacity to manage the software; Lack of unique patient identifiers among most African economies; Poor internet and power capacities; Lack of standard management; and resistance to system use by service providers among others. Thus, in the future, we expect more collaborations and partnerships to address listed challenges. For this solution to scale and achieve impact; strong, country-led partnerships are encouraged among governmental systems. African governments are encouraged to focus on effective capacity building, sustainable business model, and rigorous M&E frameworks to advance and scale health sector digitization. In addition, DHP implementers should work concertedly with the government to shape national healthcare ecosystems that connect multiple healthcare journeys, adapt innovative technologies and aggregate robust data systems into a streamlined cohesive whole to advance Regional health goals.

## Author Contributions

SI, DM, HK, and JO conceived, coordinated, and wrote the first draft of the manuscript. KG and NC participated in the study conception and overall study coordination. SI, DM, and KG contributed in writing the subsequent drafts of the manuscript. NC, HK, and JO did the final review and edit of the draft manuscript. All authors read and approved the final draft of the manuscript before publication and contributed to the article and approved the submitted version.

## Conflict of Interest

HK and JO were employed by World Health Organization – Africa Regional Office. DM was employed by World Health Organization. KG was employed by World Bank Group. NC was employed by World Bank Group. The remaining author declares that the research was conducted in the absence of any commercial or financial relationships that could be construed as a potential conflict of interest.

## Publisher's Note

All claims expressed in this article are solely those of the authors and do not necessarily represent those of their affiliated organizations, or those of the publisher, the editors and the reviewers. Any product that may be evaluated in this article, or claim that may be made by its manufacturer, is not guaranteed or endorsed by the publisher.

## References

[1] World Health Organization & International Telecommunication Union. Digital Health Platform Handbook: Building a Digital Information Infrastructure (Infostructure) for Health. World Health Organization (2020). Retrieved from: https://apps.who.int/iris/handle/10665/337449 (accessed August 11, 2021).

[2] World Health Organization. Digital Implementation Investment Guide (DIIG): Integrating Digital Interventions into Health Programmes. (2020). Retrieved from: https://www.who.int/publications/i/item/9789240010567 (accessed July 12, 2021).

[3] Atun R Jongh T Secci F Ohiri K Adeyi O. Integration of targeted health interventions into health systems: a conceptual framework for analysis. Health Policy Plan. (2018) 25:104–11. 10.1093/heapol/czp055 19917651

[4] Ibeneme S Moses O Ukor N Okeibunor J. Realigning health systems strategies and approaches; what should the African health systems do to strengthen health systems for UHC and the SDGs? Front Public Health. (2020) 8:372. 10.3389/fpubh.2020.00372 32850595 PMC7426464

[5] The Mobile Economy. Sub-Saharan Africa. GSM Association. The Mobile Economy Sub-Saharan Africa. (2020). Retrieved from: https://www.gsma.com/mobileeconomy/wp-content/uploads/2020/09/GSMA_MobileEconomy2020_SSA_Eng.pdf (accessed December 21, 2021).

[6] WHO Regional Committee,. Framework for Implementing the Global Strategy on Digital Health in the WHO African Region. (2021). Retrieved from: https://www.afro.who.int/sites/default/files/2021-11/AFR-RC71-10% 20Framework%20for%20implementing%20the%20Global%20strategy%20on %20digital%20health%20in%20the%20WHO%20African%20Region.pdf (accessed February 27, 2022).

[7] WHO Guideline: Recommendations on Digital Interventions for Health System Strengthening. Geneva: World Health Organization (2019). 31162915

[8] Ibeneme S Revere L Hwang l Rajan S Okeibunor J Muneene D. Impact of information and communication technology diffusion on HIV and tuberculosis health outcomes among African health systems. Int J Informat. (2020) 7:11. 10.3390/informatics7020011

[9] Gross N, Byers, V, Geigers, S,. Digital Health's Impact on Integrated Care, Carer Empowerment Patient-Centeredness for Persons Living With Dementia. (2021). Retrieved from: https://www.sciencedirect.com/science/article/abs/pii/S2211883721000745 (accessed March 03, 2022).

[10] Aerts N Bogdan-Martin D. Leveraging data and AI to deliver on the promise of digital health. Int J Med Inform. (2021) 150:104456. 10.1016/j.ijmedinf.2021.104456 33866232

[11] Ngoc CN Bigirimana N Muneene D Bataringaya JE Barango P Eskandar H. Conclusions of the digital health hub of the Transform Africa Summit: strong government leadership and public-private-partneship are key prerequisites for sustainable scale up of digital health in Africa. BMC J Proc. (2018) 12:17. 10.1186/s12919-018-0156-3 30540290 PMC6117634

[12] Olu O Muneene D Bataringaya JE Nahimana M Ba H Turgeon Y. How can digital health technologies contribute to sustainable attainment of universal health coverage in Africa? A perspective. Front Public Health. (2019) 7:341. 10.3389/fpubh.2019.00341 31803706 PMC6873775

[13] Moore C Werner L BenDor AP Bailey M Khan N. Accelerating harmonization in digital health. J Health Population. (2021) 17:43–54. 10.12927/whp.2017.25306 29400273

[14] Makri A,. Bridging the Digital Divide in Healthcare. (2019). Retrieved from: www.thelancet.com/digital-health (accessed March 07, 2022).

[15] Gleiss A Lewandowski S. Removing barriers for digital health through organizing ambidexterity in hospitals. J Public Health. (2022) 30:21–35. 10.1007/s10389-021-01532-y

[16] HIS Stages of Continuous Improvement Toolkit. Chapel Hill, NC: MEASURE Evaluation (2019). Retrieved from: https://www.measureevaluation.org/his-strengthening-resourcecenter/his -stages-of-continuous-improvement-toolkit/ (accessed October 23, 2021).

[17] World Health Organization PATH. Planning an Information Systems Project: A Toolkit for Public Health Managers. Seattle, WA: PATH (2013). Retrieved from: https://path.org/resources/planningan-information-systems-project-a-toolkit-for-publichealth-managers/ (accessed November 22, 2021).

[18] World Health Organization. Leave No One Behind: Strengthening Health Systems for UHC and the SDGs in Africa. (2017). Retrieved from: https://www.afro.who.int/sites/default/files/2017-12/UHC%20framework _eng_2017-11-27_small.pdf (accessed December 16, 2021).

[19] Ibeneme S Revere L Okeibunor J Hwang L Muneene D Nkiruka. Conclusions of the capacity building workshop (2019): building capacity among digital health leaders in charge of leading the development and implementation of national digital health programs in Nigeria. BMC Proc. (2020) 14:9. 10.1186/s12919-020-00193-1 32714444 PMC7376631

[20] Joshi, N,. Programming ASP.NET MVC 5 A Problem Solution Approach. Retrieved from: https://www.c-sharpcorner.com/UploadFile/EBooks/11112013031641AM/PdfFile/Programming%20ASP.NET%20MVC%205.pdf (accessed February 27, 2022).

[21] Waugaman A. From Principle to Practice: Implementing the Principles for Digital Development. (2016). Retrieved from https://digitalprinciples.org/wp-content/uploads/From_Principle_to_ Practice_v5.pdf (accessed November 12, 2021).

[22] Swanson RC Cattaneo A Bradley E Chunharas S Atun R Abbas KM. Rethinking health systems strengthening: key systems thinking tools and strategies for transformational change. Health Policy Plann. (2012) 27:54–61. 10.1093/heapol/czs090. 10.1093/heapol/czs090 23014154 PMC3529625

[23] Reimagining Global Health through Artificial Intelligence: The Roadmap to AI Maturity. Broadband Commission for Sustainable Development (2020). Retrieved from: https://www.novartisfoundation.org/sites/novartisfoundation_org/files/2020-12/reimagining-global-health-through-artificial-intelligence-a-roadmap-to-ai-maturity.pdf (accessed October 8, 2021).

[24] Orem NJ, Karamagi, H, Omar, S, Tumusiime, P,. WHO Africa's Third Forum on Health Systems Strengthening for UHC the SDGs. (2018). Retrieved from: http://www.internationalhealthpolicies.org/who-africas-third-forum-on -health-systems-strengthening-for-uhc-and-the-sdgs (accessed November 23, 2021).

[25] World Health Organization. WHO Digital Documentation of COVID-19 Certificates: Vaccination Status (DDCC:CC). (2021). Retrieved from: https://WorldHealthOrganization.github.io/ddcc (accessed December 21, 2021).

[26] Meyer C, Kim, E, Husain, I,. Developing Economic Impact Assessment Methods to Identify the Costs of Artificial Intelligence-Driven Health Technology. United States Agency for International Development Policy Paper. (2021). Available online at: https://pdf.usaid.gov/pdf_docs/PA00XGXF.pdf (accessed November 11, 2021).

[27] World Health Organization. Classification of Digital Health Interventions: A Shared Language to Describe the Uses of Digital Technology for Health. Geneva: World Health Organization (2018). Retrieved from: https://www.who.int/reproductivehealth/publications/mhealth/classificationdigital-health-interventions/en/ (accessed October 24, 2021).

[28] Uohara MY Weinstein JN Rhew DC. The essential role of technology in the public health battle against COVID-19. Popul Health Manag. (2020) 23:361–7. 10.1089/pop.2020.0187 32857014

